# Localization of Mineralocorticoid Receptors at Mammalian Synapses

**DOI:** 10.1371/journal.pone.0014344

**Published:** 2010-12-15

**Authors:** Eric M. Prager, Jennifer Brielmaier, Hadley C. Bergstrom, Jennifer McGuire, Luke R. Johnson

**Affiliations:** 1 Program in Neuroscience, Uniformed Services University, Bethesda, Maryland, United States of America; 2 Department of Psychiatry, Uniformed Services University, Bethesda, Maryland, United States of America; 3 Center for the Study of Traumatic Stress, Uniformed Services University, Bethesda, Maryland, United States of America; Cuban Neuroscience Center, Cuba

## Abstract

In the brain, membrane associated nongenomic steroid receptors can induce fast-acting responses to ion conductance and second messenger systems of neurons. Emerging data suggest that membrane associated glucocorticoid and mineralocorticoid receptors may directly regulate synaptic excitability during times of stress when adrenal hormones are elevated. As the key neuron signaling interface, the synapse is involved in learning and memory, including traumatic memories during times of stress. The lateral amygdala is a key site for synaptic plasticity underlying conditioned fear, which can both trigger and be coincident with the stress response. A large body of electrophysiological data shows rapid regulation of neuronal excitability by steroid hormone receptors. Despite the importance of these receptors, to date, only the glucocorticoid receptor has been anatomically localized to the membrane. We investigated the subcellular sites of mineralocorticoid receptors in the lateral amygdala of the Sprague-Dawley rat. Immunoblot analysis revealed the presence of mineralocorticoid receptors in the amygdala. Using electron microscopy, we found mineralocorticoid receptors expressed at both nuclear including: glutamatergic and GABAergic neurons and extra nuclear sites including: presynaptic terminals, neuronal dendrites, and dendritic spines. Importantly we also observed mineralocorticoid receptors at postsynaptic membrane densities of excitatory synapses. These data provide direct anatomical evidence supporting the concept that, at some synapses, synaptic transmission is regulated by mineralocorticoid receptors. Thus part of the stress signaling response in the brain is a direct modulation of the synapse itself by adrenal steroids.

## Introduction

The amygdala, particularly the lateral nucleus, (LA) is established as a site for the acquisition and storage of conditioned fear memory and a trigger of the stress response [1]–[5]. The expression of conditioned fear includes amygdala dependent activation of the hypothalamic-pituitary-adrenal (HPA) axis. The HPA-axis increases blood concentration of corticosterone in rodents, which in turn feeds back to the brain including the amygdala where it binds to both mineralocorticoid (MRs) and glucocorticoid receptors (GRs) [6]–[8]. In the hippocampus, MR and GR activation elicits both rapid, non-genomic effects [8]-[10] resulting in changes to synaptic transmission. Cytosolic MR and GR also translocate to the nucleus upon activation, which results in changes in gene transcription [11]–[13]. The cellular mechanisms of MR and GR signaling in the LA are not fully understood. However, recent evidence suggests the LA likely contains signaling mechanisms that include both classic nuclear MR and GR signaling as well as direct signaling from the synapse via membrane receptors mechanisms [7], [8], [14], [15], which regulates the fear response and influence fear related behaviors [16]–[22].

Both MR and GR regulate the acquisition and consolidation of fearful memories and may be implicated in stress related disorders [4], [23]–[26]. Behavioral evidence suggests that activation of the MR and GR may modulate the acquisition and consolidation of fear memories [8], [18], [27]–[33]. Membrane MR (mMR) and GR (mGR) may also be implicated in the formation of fear memories. Corticosterone (CORT) conjugated to a high molecular weight substance (e.g., bovine serum albumin; BSA) [34] prevents CORT from entering the cell and can be used to study the nongenomic activity of mMR and mGR [8], [10], [35]–[39]. Activating mMR and mGR via CORT-BSA is known to induce cognitive deficits including impairing working memory [40]. Rapid elevations in CORT are also known to impair successful encoding of new information [41], [42], retention [43] and consolidation [44], and the retrieval of a long-term memory [45].

Electrophysiological evidence suggests the presence of a rapid membrane MR and GR at both pre- and postsynaptic locations [8]–[10], [46]–[53], which rapidly induce changes to synaptic transmission. Moreover, behavioral and cognitive evidence suggests the presence of both MR and GR throughout the limbic system, including the amygdala. While the presence of MR and GR has been examined in the hippocampus [54], [55] to date only one study has anatomically described the GR in the amygdala and in LA synapses [7] in adrenal cortex intact animals. No study has examined the anatomical expression of MR in the LA or in LA synapses. The purpose of this experiment is to extend available evidence of GR expression in LA synapses and to provide the first anatomical evidence that endogenous MR are expressed in LA neurons and at the synapse. Results from the data described suggest the presence of MR at LA synapses placing them in position to regulate synaptic plasticity underlying the acquisition of fear memories.

## Results

### Western Blot Immunoassay

Western blot immunoassay was used to determine the presence of MR in amygdala nuclei. Two control regions were also included. First, tissue from the hippocampal formation was included because it has previously been reported to contain MR [56]–[58]. Second, separate tissue from several hypothalamic subnuclei was tested because it has previously been reported to contain very low to undetectable MR [56]. Thus the hippocampus and hypothalamus served as high and low MR control regions, respectively. Additionally, the specificity of antibodies including any potential cross-reactivity used in subsequent immuno electron microscope studies, to MR and GR, was confirmed with the immunoassay.

**Figure 1:**
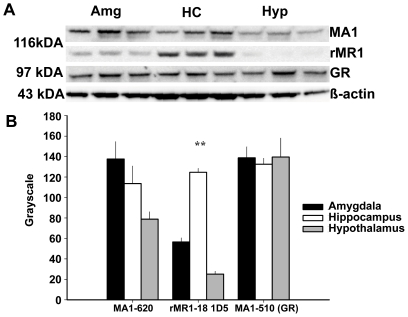
Immunoblots of MR and GR in amygdala (Amg), hippocampal (HC) and hypothalamic (Hyp) nuclei. As indicated by the representative immunoblots standardized to β-Actin (A) there was a significant difference in relative density of MR between brain nuclei (B). GR was equally distributed in all brain regions. (** denotes p's<.01).

Immunoassay results revealed the presence of MR in amygdala tissue. No cross-reactivity of labeling between the GR and MR was observed. The GR and MR labeling consisted of single bands at the molecular weights of 116 or 97 kDA, respectively (Fig. 1A). A two-way ANOVA revealed an interaction of labeled receptors by brain region for receptor labeling (F (4, 18) = 7.910, p = 0.001). Subsequent post hoc comparison of the amygdala versus hypothalamus for the antibody MA1-620 suggests a statistical trend towards a greater intensity of labeling in the amygdala (p = .065). There was no difference in the intensity of MR labeling between the amygdala and hippocampus. A second MR antibody that binds to the first 18 amino acids of the receptor (rMR1-18 1D5) was compared with the original antibody, which binds between amino acid sequence 770 and 945. The intensity of labeling in the amygdala was greater than in the hypothalamus (p = 0.002) but less than in the hippocampus (p<.001). No differences in GR were found between any of the brain regions (Fig. 1B). These data suggest initial evidence that MR are expressed in amygdala nuclei and that the there was no cross-reactivity between antibodies.

### Light Microscopy

**Figure 2:**
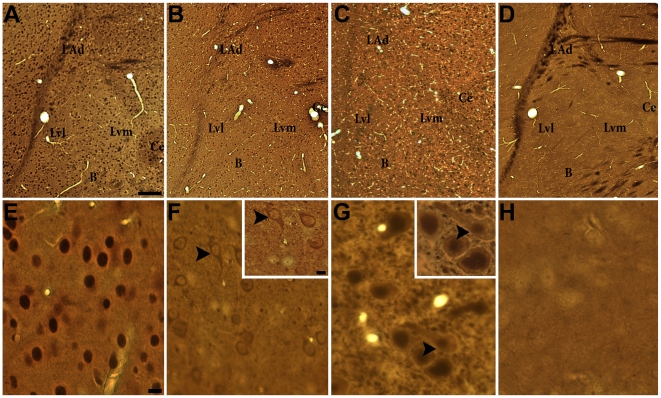
GR and MR labeling in the rat amygdala. (A & E) GR-ir neurons are distributed throughout the amygdala predominately labeling the nucleus. (B & F) MR-ir (MA1-620) neurons are distributed throughout the amygdala but immunoreactivity is not as dense as GR-ir. Large proximal dendrites showed immunoreactivity (F inset). (C & G) MR-ir (rMR1-18 1D5) neurons are distributed in amygdala nuclei and cell membrane but do not appear as densely labeled in perikaryon (G inset). (D & H) Control section shows little immunoreactivity of cells in amygdala or surrounding nuclei. Scale bars = 200 µm for A–D, 10 µm for D–G and 5 µm for F & G insets. Amygdala nuclei subdivisions B = basal, Ce = central, Lad = lateral (dorsal division), Lvl = lateral (ventral-lateral), Lvm = lateral (ventral-medial).

Amygdala neuron immunoreactivity (ir) was qualitatively examined using the light microscope to confirm the immunoassay results. GR-ir and MR-ir was revealed in cells throughout the amygdala sub-regions, including lateral amygdala (LA), the basal nucleus (B), and central nucleus (Ce), compared to control sections. GR-ir was observed at the nucleus of principal and inhibitory LA cells (Fig. 2A and 2E) but also in small dendrites and apparent spines. In contrast, the MR-ir labeled with the MA1-620 antibody (Fig. 2B and 2F) were more dispersed and appeared less dense than GR nuclei. MR-ir was predominately found in perikaryon, the nucleolus, proximal dendrites, and in apparent dendritic spines (Fig. 2F inset). MR-ir labeled with the rMR1-18 1D5 [55] antibody revealed MR scattered throughout both the nucleus and the cell membrane (Fig. 2C and 2G). Peripherally, MR also appeared in neuropil, proximal dendrites and apparent dendritic spines (Fig. 2G inset).

**Figure 3:**
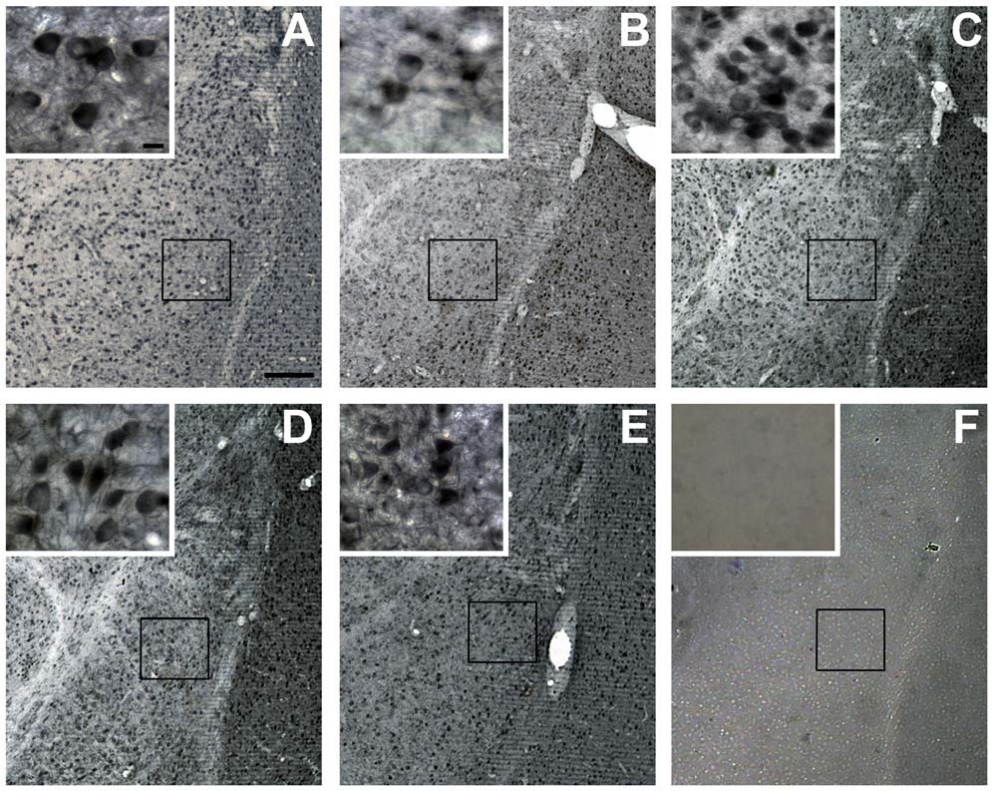
Preabsorbing antibodies with aldosterone or a peptide reduces or blocks visualization. MR-ir controls were established to ensure specificity of the antibodies. MA1-620 (A) and rMR1-18 1D5 (D) were visualized using SG chromogen. Tissue was incubated with 1 µMol aldosterone 30 min before addition of primary antibody. Aldosterone reduced observable chromogen visualization in neurons in the LA (B) when incubated with the antibody MA1-620. No observable difference was found when incubating aldosterone in tissue with the antibody rMR1-18 1D5 (E). Incubating the peptide used to generate the second antibody with the MA1-620 antibody (C) did not produce observable differences in chromogen visualization. Incubating the peptide in tissue with the antibody rMR1-18 1D5 completely blocked the antibody from binding to tissue (F). Scale bars = 200 µm for A–F, 5 µm for insets.

We ran additional control analyses using SG chromogen labeling. The groups consisted of aldosterone (1 µMol) applied to the antibody MA1-620 or applied to the antibody rMR1-18 1D5, and the peptide (METKGYHSLPEGLDMERR) used to generate the antibody rMR1-18 1D5 applied to both MR antibodies. We qualitatively examined tissue preabsorbed with the peptide or aldosterone and compared this with tissue incubated in the primary antibody alone (Fig. 3A and 3D). Tissue incubated with aldosterone and the antibody MA1-620 had the appearance of reduced labeling. Using Image J (National Institutes of Health, Bethesda, MD), we quantified the number of cells labeled in each condition. Tissue sections incubated in aldosterone (n = 4) (Fig. 3B) were quantitatively compared with sections incubated in the antibody MA1-620 (n = 3) (Fig. 3A). A significant reduction in MR labeling was found when tissue was first incubated in 1 µMol aldosterone (t = 3.341; p = 0.021). Although a significant decrease in labeling was determined, there appeared to be a degree of nonspecificity for the antibody (Fig. 3B), which may be because the antibody was generated from aldosterone-3 and not generated from the receptor itself. Yet both Western blot data suggest labeling is specific to the MR. We next incubated the antibody MA1-620 with the peptide used to generate the second MR antibody (rMR1-18 1D5) (Fig. 3C). We observed no difference in labeling from the positively labeled MA1-620 tissue, providing further evidence that the antibody MA1-620 binds to a different epitope than that of the antibody rMR1-18 1D5.

Qualitative analysis of the antibody rMR1–18 1D5 was also examined. Compared with tissue incubated in the primary antibody alone (Fig. 3D), tissue preabsorbed in aldosterone did not reduce labeling (Fig. 3E), providing further evidence of specificity of the antibodies. However, preabsorbing the antibody rMR1–18 1D5 with the peptide used to generate this antibody completely blocked the antibody from binding to receptors in the tissue (Fig. 3F). Together the data revealed that tissue incubated with excessive agonist or the peptide used to generate the antibodies reduced or prevented observable labeling in their respective antibodies but not in the opposing antibody, suggesting specificity of the antibodies to the receptors.

### GR and MR Labeling in Nuclear and Extra Nuclear Structures

**Figure 4:**
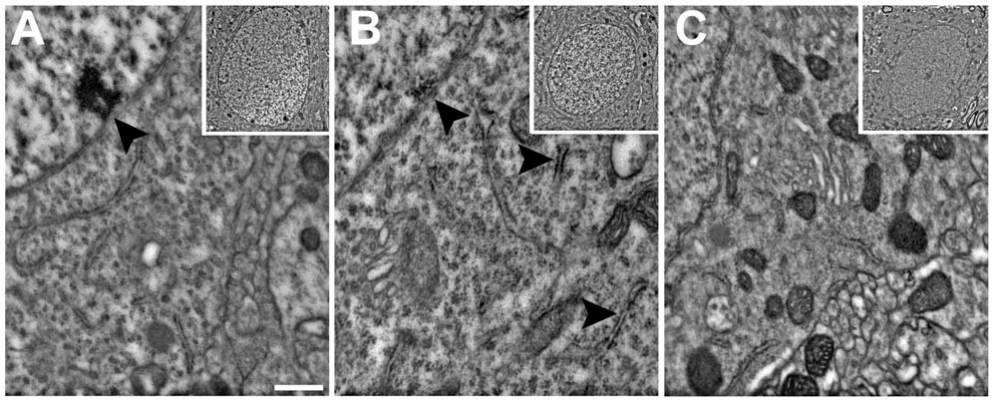
GR and MR immunoreactivity in the nucleus and perikaryon. (A) Supporting light level qualitative analysis, immunoreactivity was observed in the nuclear compartment of GR-ir labeled LA neurons and (B) MR-ir (rMR1-18 1D5) labeled neurons compared to primary antibody omitted control slices (C). Little immunoreactivity was observed in the nucleus of LA neurons labeled with the MR antibody MA1-620. MR immunoreactivity with both antibodies was observed on cytosolic structures including the Golgi Apparatus and cell membrane (B). Scale bars = 500 nm.

**Figure 5:**
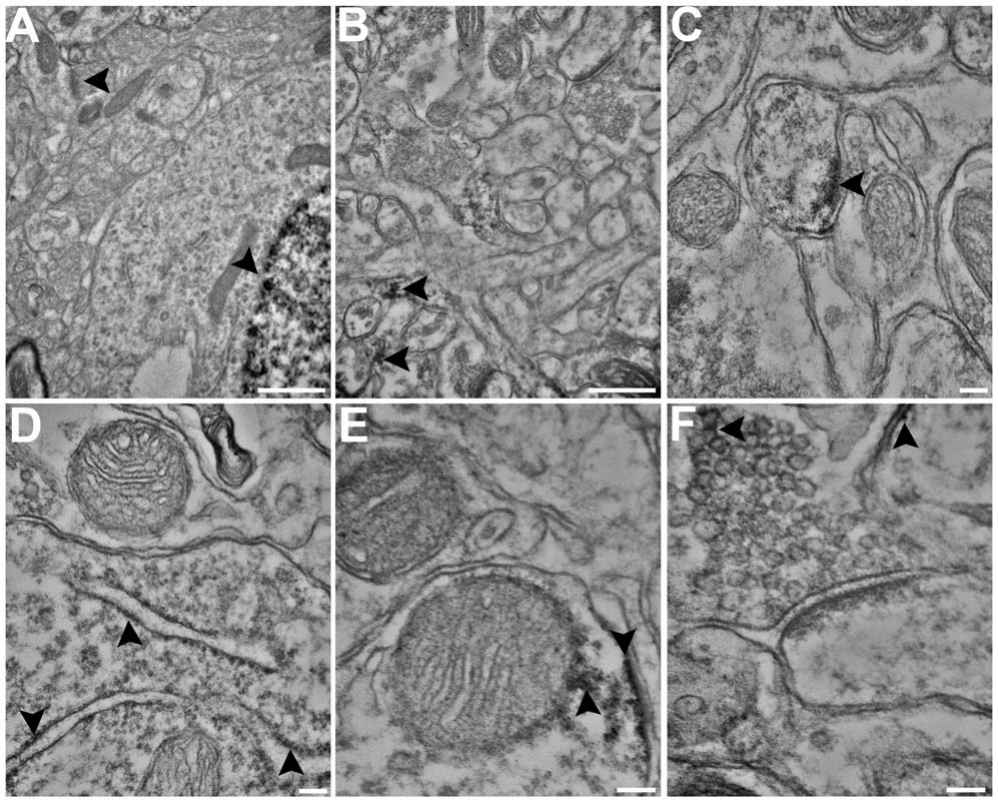
MR-ir in extra nuclear structures. A significant difference between MR-ir with both antibodies and primary antibody omitted control sections were observed in multiple extra nuclear structures. Although not observed as frequently as principal neuronal structures, GABAergic interneurons were observed to have immunoreactivity (see comparison with an unlabeled asymmetrical synapse) (A). Glial processes (B), dendritic membranes (C), Golgi apparatus (D), mitochondrial membranes (E), and presynaptic vesicles (F) also were observed to have immunoreactivity compared with respective primary antibody omitted controls. Scale bars = A–B 500 nm, C–F = 100 nm.

Nuclear and perikaryon MR-ir specificity was examined after confirming the presence of MR in amygdala neurons. Principal neurons (Fig. 4A–C), GABAergic interneurons (Fig. 5A), and glial processes in the LA were observed using the transmission electron microscope (Fig. 5B and 5C). Cell types show characteristics of GR- and MR-immunoreactivity when compared to controls. Confirming the light microscopy examination, GR-ir was expressed more in the nucleus than in the perikaryon. MR-ir neurons had nuclear labeling with the antibody rMR1–18 1D5, but not MA1–620 (Fig. 4 and 5A).

Primary antibody omitted control sections were compared with MR-ir and GR-ir sections and contained little immunoreactivity in the nucleus or cytosolic organelles. GR-ir was observed on ribosomes, Golgi apparatus, mitochondria, presynaptic terminals, and the PSD (Fig. 5B–F). Similarly, MR-ir (with both MA1–620 and rMR1–18 1D5) was observed on ribosomes and Golgi apparatus, mitochondria, the cellular membrane, postsynaptic density (PSD), and on both the presynaptic terminal and vesicles (Fig. 5F). Control sections revealed little immunoreactivity in the nucleus or cytosolic organelles, suggesting that the primary antibody labeling was specific to the GR or MR. In sum, glutamatergic and GABAergic neurons both contained MR-ir and GR-ir in nuclear and non-nuclear structures. MR-ir appeared primarily on neuronal cells and appeared to primarily be expressed in dendrites and in spines but also, with the antibody rMR1-18 1D5 in the nucleus.

### GR and MR Labeling of Asymmetrical Synapses

**Figure 6:**
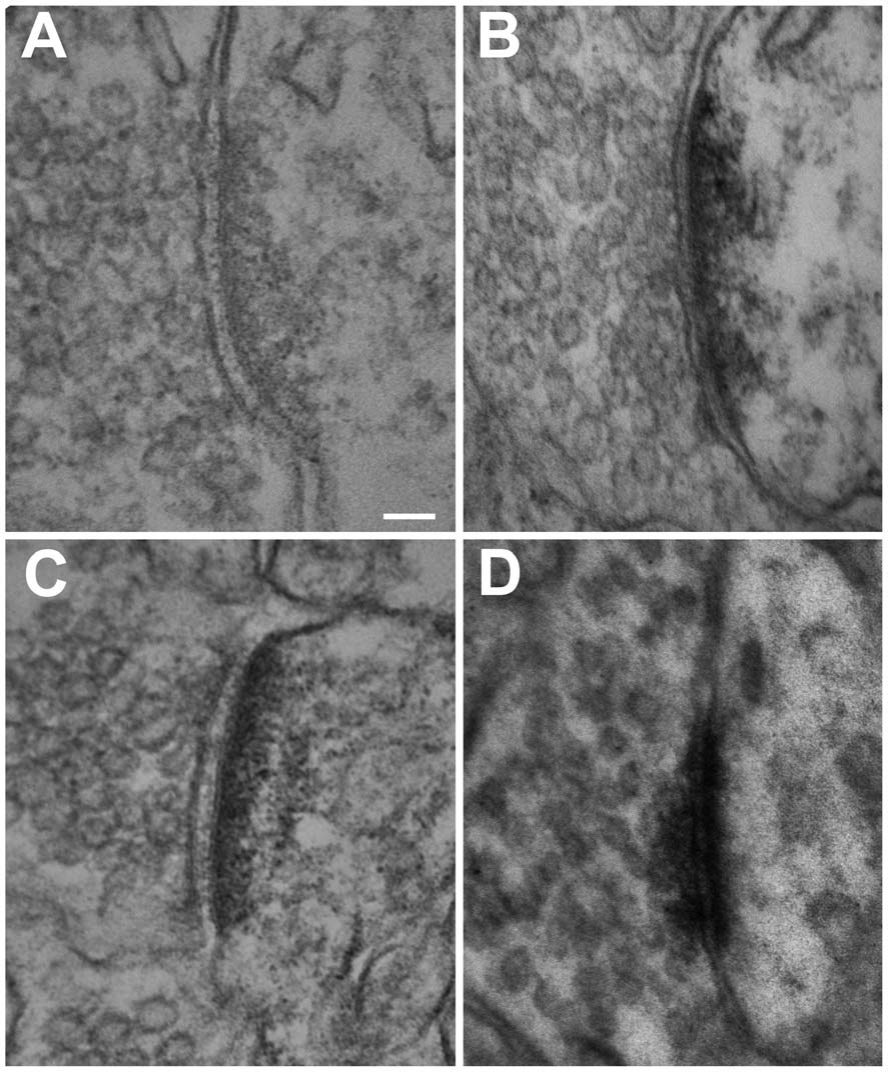
GR and MR labeling at asymmetrical synapses. Asymmetrical synapses were examined for immunoreactivity since electrophysiological evidence has examined excitatory synaptic responses of MR and GR. Compared with primary antibody omitted control asymmetrical synapses (A), immunoreactivity was observed in the postsynaptic density of GR-ir LA neurons (B) and MR-ir (MA1–620; rMR1–18 1D5) LA neurons (C and D, respectively). MR-ir was also observed at presynaptic terminals of asymmetric synapses (D). Scale bar = 100 nm.

MR-ir and GR-ir was expressed in pre- and postsynaptic sites. Qualitative examination of asymmetrical synapses revealed both GR-ir and MR-ir (MA1–620 and rMR1–18 1D5) at the PSD (Fig. 6B–D) and MR-ir at the presynaptic terminal (Fig. 6D), compared to the primary antibody omitted PSD (Fig. 6A).

**Figure 7:**
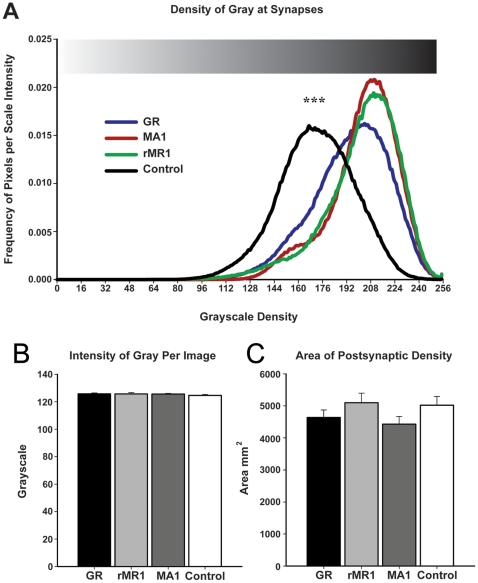
Quantitative analysis of labeling at postsynaptic density of asymmetrical synapses. MR-ir and GR-ir at asymmetrical synapses was determined using a relative measure of density of pixels at individual asymmetrical synapses. GR-ir and MR-ir labeled asymmetrical synapses had significantly greater immunoreactivity compared with primary antibody omitted control sections, suggesting the presence of the receptors at the synapse (A). No differences were found between the two MR-ir groups nor between the MR and GR groups. Two control analysis were utilized to ensure that the differences in intensity of labeling at the synapses were not due to the intensity of gray of images taken (B) nor to the size of the PSD (C). (*** depicts p<.001).

A quantitative analysis of MR-ir and GR-ir at the PSD was performed to determine whether immunoreactivity was different from primary antibody omitted control sections using a relative measure of gray intensity (Fig. 7A). Two control analyses were ran to establish that differences in gray intensity at the PSD were due to the DAB and not influenced by the electron beam intensity or size of the PSD. First, the gray intensity of each individual image was examined to ensure an equal distribution of gray. Indirectly, this also ensured that the intensity of the electron beam was consistent across each picture. A one-way ANOVA revealed no significant differences between each condition (p = .554) (Fig. 7B). The area of the PSD was then examined to ensure that there were no differences in the size of the area measured. As predicted, there were no significant differences in PSD size between any of the groups (p = .219) (Fig. 7C).

Asymmetrical synapses (n = 250) examined across the four conditions revealed a significant difference between labeled and unlabeled synapses (F = 39.09; p<.001). Planned contrasts between the groups revealed a significant difference between the primary antibody omitted control and both MRs and GR (t = 10.496; p<.001). No difference between GR and either of the MR groups (MA1–620 (p = 0.61); or rMR1–18 ID5 (p = 0.77) (Fig. 7A) was found. There was no significant difference between the two MR groups (p = .775). Overall, these data provide quantitative evidence that both MR and GR are in asymmetrical synapses of LA neurons.

## Discussion

This study provides the first anatomical evidence for the expression of MR in LA synapses. The Western blot immunoassay data established the presence of MR in amygdala neurons and ensured no cross-reactivity between antibodies and receptors. Examination of immunoreactivity using the electron microscope provided evidence for expression of MR to the nucleus and extra nuclear sites including synapses of LA neurons. MR-ir was also observed in glial processes, and in cellular protein generation and cell respiration machinery including ribosomes, mitochondria, and Golgi apparatus. MR-ir was observed in the presynaptic terminal (Fig. 6D), on vesicles (Fig. 5F), and within the PSD itself (Fig. 6C and 6D). Expression of the MRs at both the presynaptic terminal and the PSD suggest MRs may be positioned to regulate glutamate release from the presynaptic terminal and also to regulate post synaptic responses, both of which has been demonstrated by recent electrophysiological studies [10], [52]. These data support a growing body of evidence for direct regulation of synaptic transmission during stress mediated by adrenal hormone signaling [5], [8], [47], [49], [51], [59].

The classical signaling pathway of hormone receptors is to translocate into the nucleus where they homodimerize and bind to hormone response elements thereby modulating gene transcription [8], [51], [60], [61]. Evidence from studies in neurons suggests gonadal and adrenal hormone receptors are also membrane associated and have an additional signaling mechanism, which is nongenomic and may involve regulation of ion channels and second messengers systems at membrane and especially synaptic sites [8], [14], [51]. For example, the estrogen receptor was first characterized to have membrane-bound properties in the 1970s by Pietras and Szeko [62], [63]. Likewise, the androgen [64], progesterone, [65], [66], and glucocorticoid [7] receptors have all been previously characterized as membrane associated hormone receptors. This study is the first to anatomically demonstrate that the MRs are also membrane associated.

In order to confirm MR was expressed in the PSD, two antibodies, which recognize different epitopes of the MR, were used to visualize the presence of the receptor throughout neurons and glia of the LA. The MA1-620 antibody recognizes the ligand-binding site of the MR. When examining the tissue for MR labeling at the light microscope level, MA1–620 labeled receptors that were primarily cytosolic (Fig. 2 and Fig. 3). MR-ir was significantly reduced with the application of 1 µMol aldosterone (Fig. 3B). The rMR1–18 1D5 antibody recognizes a different binding site (see Fig. 3D–F) and labeled receptors in the nucleus, perikaryon, and in neuropil (Fig. 3). The difference in labeling may be because aldosterone and corticosterone competes for the ligand-binding site and will displace the antibody if present. Alternatively, because rMR1–18 1D5 binds to a different epitope (amino acids 1–18), both active and inactive MR was observed. MR labeling using both antibodies was also investigated at the electron microscope level.

MR labeling, as observed in the electron microscope using both antibodies, was consistent with a fast acting nongenomic mode of action of the receptors. Both MA1–620 and rMR1–18 1D5 revealed consistent labeling of the synaptic PSD. Moreover, quantitative gray scale measures of relative intensity of labeling were not significantly different between antibodies (Fig. 7). This suggested that at the synapse, receptor recognition by the different antibodies was not affected by the endogenous ligand. The degree to which the synaptic MR and are regulated by circulating corticosterone levels in both intact and adrenalectomized animals is an important question for future research.

Studies of MR DNA knockout mice suggest that mMR are encoded by the same genes as their genomic counterparts [10]. However these receptors may undergo a posttranslational modification that results in membrane translocation [8], [67]. Although it remains unknown how the MR becomes membrane bound, the GR appears to be covalently modified by the insertion of a long chain fatty acid, most notably palmitic acid [68], [69]. Palmitoylation is a covalent attachment of long-chain fatty acids that increase protein hydrophobicity and membrane association of proteins [70], such as the estrogen receptor and GR. Recent analysis [67], [69], [71], [72] suggests that the palmitoylation sequence is recognized in most steroid receptors, including the GR, progesterone, estrogen, and the androgen receptor. If the nuclear receptors are modified by the lipid moiety, then it is entirely possible that the membrane receptors are the same as their nuclear counterparts and function to regulate synaptic excitability.

Although biochemical isolation of mMR remains unconfirmed, electrophysiological evidence suggests functional synaptic expression (see [8], [14] for thorough reviews). Activation of mMR by low doses of corticosterone increase the release of glutamate from presynaptic terminals [10], [52] through an extracellular-signal regulated kinase (ERK) pathway, while at the same time inhibiting potassium efflux postsynaptically [52]. Fast acting responses after application of corticosterone has also been found to increase GluRII subunit motility of the AMPA receptor and may either enhance or suppress LTP [9], [73], [74].

Memory acquisition may be differently regulated by mMR and mGR in the amygdala compared to the hippocampus during an acute stress. However underlying differences in mechanisms are not understood. Electrophysiological evidence suggests mMR enhances whereas mGR inhibits synaptic transmission in the hippocampus. The activation of the mMR and mGR may be dependent on the concentration of corticosterone. An inverted U-shaped dose response has been proposed to describe the relationship between activation of mMR and mGR and changes in neuronal excitability [8]. Less characterized is the nongenomic response of the mMR and mGR in the amygdala. Recent electrophysiological evidence from the LA suggests greater mMR relative to mGR activation might modulate AMPA receptor subunit (GluRII) motility and current [9], [73], [74]. Although scarce, results from behavioral studies suggest that activating mMR and mGR impair working and may impair the consolidation of a fear memory [17], [40], [75].

In summary, MR and GR expression in the LA was examined because the LA is the site of fear memory formation [1], [3], [76] and activated mGR and mMR may regulate the acquisition of that memory [4], [7], [8], [16], [17]. MRs were expressed at both nuclear and extra-nuclear LA structures, including the synapse. These data are in support of electrophysiological evidence suggesting MR are functionally positioned to regulate synaptic excitability at synapses and, in turn, the acquisition of fear memories [5], [8], [17], [18], [30], [77].

## Materials and Methods

### Animals

20 male Sprague-Dawley rats with intact adrenal glands were used in this study (approximately 275–350 g; Taconic Farms, Hudson, NY). Animals were left with intact adrenal glands in order to obtain a non-manipulated subcellular anatomical profile for MR and GR. This has been commonly used in both behavioral [7], [41], [42], [78] and electrophysiological [10], [36], [37] studies. All animals were group housed for 6 days prior to experimentation with food and water provided *ad libitum*. Lights were maintained on a normal light cycle (07:00 until 19:00 h) and the temperature and humidity were kept at 24°C and 44%, respectively. All animal procedures related to maintenance and experimentation were in accord with the National Institutes of Health guidelines and approved by the Institutional Animal Ethics Committee.

### Western Blot Analysis

Three male Sprague-Dawley rats were used in Western Blot immunoassay analysis. For isolation of total proteins, 25–50 mg samples of amygdala, hippocampus, and hypothalamus from each rat were crushed with mortar and pestle in dry ice and dispersed with a sonicator (Kontes micro-ultrasonic cell disrupter) in lysis buffer (10×PBS, 50 mL; 10% SDS, 5 mL; NP-40, 5 mL; sodium deoxycholic acid, 2.5 g; and double-distilled water to 500 mL) containing 1×TBS, 0.1% Triton-X-100, and protease inhibitors (Complete Protease Inhibitor Cocktail Tablets; Boehringer-Mannheim, Germany). Lysates were centrifuged at 12,000 rpm for 20 min at 4°C. Total protein concentrations were determined according to manufacturer's instructions (Protein Assay Kit; BioRad).

Twenty-five micrograms of total protein from each sample were boiled for 5 min then loaded on NuPage Novex 4–12% Bis Tris gels (Invitrogen, Carlsbad, CA) and separated electrophorectically. Proteins were transferred to nitrocellulose membranes with the I-Blot (Invitrogen, Carlsbad, CA). Nitrocellulose membranes were incubated in blocking buffer (5% dry nonfat milk in Tris-buffered TBS (TBST): 20 mM Tris-HCL, pH 7.4, 150 mM NaCl, and 0.1% Tween-20) for 1 h followed by incubation in primary antibodies against the GR (1∶200, MA1–510 from Affinity Bioreagents, Golden, CO) and MR (MA1–620 (1∶1000) from Affinity Bioreagents and rMR1–18 1D5 (1∶200) [55], Developmental Studies Hybridoma Bank, Iowa City, IA) overnight at 4°C on a shaker table. Membranes were washed in washing buffer (TBST), and then incubated with the appropriate HRP-conjugated secondary antibody (1∶5000) for 60 min at room temperature. Membranes were then washed and incubated with chemiluminescent detection reagents (Pierce, SuperSignal West Femto Maximum Sensitivity Substrate) for 5 min, and quantified with a Fuji Film LAS-1000 camera and LAS-1000plus software. Relative protein levels were determined from the optical densities of the corresponding protein bands after subtraction of background values obtained from the same lane. All protein samples were then standardized with β-actin, an internal control.

### Immunohistochemistry Analysis

One week after arrival and habituation to the environment, animals were deeply anesthetized with between 0.5 and 1.63 mL chlorohydrate. Animals were then transcardially perfused with 200 mL of physiological saline (0.9% NaCl in distilled H_2_O) over a 30 sec period followed by perfusion with 200 mL of 4% paraformaldehyde and 3% acrolein in 0.1 M phosphate buffer (PB), pH 7.4. Brains fixed for light level analysis were perfused with 200 mL of 1% glutaralydehyde (GLUT) and 4% paraformaldehyde (PFA). The brains were removed from the skull, washed with PBS, and post-fixed in 4% PFA and 3% acrolein for 60 min or 4% PFA and 1% GLUT for 6 hours. Brain slices were sectioned at 30 µm and placed into one of four conditions. Following blocking with normal horse serum (Vector Labs, Burlingame, CA) for 60 min, primary antibodies of GR (MA1–510) and two MR (MA1–620 and rMR1–18 1D5) were applied for 48 hours at concentrations of 1∶100. Additional control samples were incubated in either the peptide in which the antibody rMR1–18 1D5 was generated (5 mg/ml) or in 1 µMol aldosterone. This was followed by application of a biotinylated secondary antibody for 24 hours at a concentration of 1∶200 (Vector Labs, Burlingame, CA). Tissue sections were then incubated in PBS containing bovine serum albumin (ABC) for 60 minutes. The reaction product was visualized by incubation in 3,3′-Diaminobenzidine (DAB) and hydrogen-peroxide [7], [79], (Sigma, St. Louis, MO, USA) for 20 min. Sections prepared for light observation were visualized by incubation in SG chromogen (Vector Laboratories, Burlingame, CA) for 6–10 min, washed, dehydrated, and mounted on gelatin slides. Following application of 2% osmium tetroxide for 20 min, sections prepared for electron microscopy were post-fixed with 1% uranyl acetate in 70% ethanol, dehydrated, and placed in resin. Ultrathin sections (85 nm) were then cut from LA and collected on 6–9 nickel slot grids. For single labeling (MR-immunoreactive (ir) only or GR-immunoreactive (ir) only) the primary antibody was omitted and specificity of the secondary antibody was tested [7]. Control and experimental sections were incubated and processed for all steps in parallel.

Neuronal cell types and intracellular organelles were examined. Principal, glutamatergic neurons are morphologically characterized based on the presence of smooth non-invaginated oval nuclei with a thin rim of cytoplasm whereas inhibitory GABAergic interneurons are characterized as having a round invaginated nucleus with a thicker rim of cytoplasm [7], [80]–[83]. Somata were identified by the presence of a nucleus and endoplasmic reticulum; axon terminals were identified by the presence of synaptic vesicles. Dendrites were identified by the presence of microtubules, postsynaptic densities, and the presence of spines [79]. Dendritic spines were identified as either asymmetric (thickened PSD) or symmetric (thin or absent PSD) [84]. Glial cells were identified by irregular contours. Statistical analysis was performed using SPSS (SPSS, Chicago Il) and included analysis of variance (ANOVA).
